# Low-voltage ride-through capability in a DFIG using FO-PID and RCO techniques under symmetrical and asymmetrical faults

**DOI:** 10.1038/s41598-023-44332-y

**Published:** 2023-10-16

**Authors:** Kiomars Sabzevari, Nima Khosravi, Muhammad Bakr Abdelghany, Youcef Belkhier, Marcos Tostado-Véliz, Hossam Kotb, Scott Govender

**Affiliations:** 1 Department of Electrical Engineering, Technical and Vocational University (TVU), Tehran, Iran; 2 Department of Electrical and Instrumentation Engineering, R&D Management of NPC, Tehran, Iran; 3 Department of Electrical and Computer Engineering, Khalifa University of Science and Technology, Sas Al-Nakhl Campus, Abu Dhabi, United Arab Emirates; 4 Computer and Systems Engineering Department, Faculty of Engineering, Minia University, Minia, Egypt; 5 Institut de Recherche de l’Ecole Navale (EA 3634, IRENav), French Naval Academy, 29240 Brest, France; 6 Department of Electrical Engineering, University of Jaén, 23700 Linares, Spain; 7 Department of Electrical Power and Machines, Faculty of Engineering, Alexandria University, Alexandria, 21544 Egypt; 8 Department of Power Systems Operation and Planning, Power Electrical Industry Consultants Co, Limbe, Malawi

**Keywords:** Electrical and electronic engineering, Wind energy

## Abstract

The power grid faults study is crucial for maintaining grid reliability and stability. Understanding these faults enables rapid detection, prevention, and mitigation, ensuring uninterrupted electricity supply, safeguarding equipment, and preventing potential cascading failures, ultimately supporting the efficient functioning of modern society. This paper delves into the intricate challenge of ensuring the robust operation of wind turbines (WTs) in the face of fault conditions, a matter of substantial concern for power system experts. To navigate this challenge effectively, the implementation of symmetrical fault ride-through (SFRT) and asymmetrical fault ride-through (AFRT) control techniques becomes imperative, as these techniques play a pivotal role in upholding the stability and dependability of the power system during adverse scenarios. This study addresses this formidable challenge by introducing an innovative SFRT–AFRT control methodology based on rotor components optimization called RCO tailored for the rotor side converter (RSC) within a doubly-fed induction generator (DFIG) utilized in wind turbine systems. The proposed control strategy encompasses a two-fold approach: firstly, the attenuation of both positive and negative components is achieved through the strategic application of boundary constraints and the establishment of reference values. Subsequently, the optimization of the control characteristic ‘$\beta$’ is accomplished through the utilization of a particle swarm optimization (PSO) algorithm integrated within an optimization loop. This intricate interplay of mechanisms aims to optimize the performance of the RSC under fault conditions. To measure the efficacy of the proposed control technique, a comparative analysis is conducted. Fractional-order (FO) proportional–integral–derivative (PID) controllers are employed as an additional method to complement the novel approach. By systematically juxtaposing the performance of the proposed SFRT–AFRT control technique with the FO-PID controllers, a comprehensive evaluation of the proposed approach's effectiveness is attained. This comparative assessment lends valuable insights into the potential advantages and limitations of the novel control technique, thereby contributing to the advancement of fault mitigation strategies in WT systems. Finally, the paper highlights the economic viability of the proposed control method, suggesting its suitability for addressing broader power network issues, such as power quality, in future wind farm research.

## Introduction

Preserving the operational resilience of doubly-fed induction generator (DFIG) systems, along with fortifying the integrity of the intricate power grid infrastructure, assumes an unparalleled significance in the realm of contemporary electrical engineering. This imperative emerges from the intricate interdependence between burgeoning renewable energy sources, notably wind turbines equipped with DFIG technology, and the established fabric of the power grid. Within this symbiotic relationship, a meticulous and rigorous focus on strategies for mitigating the potential ramifications of both symmetrical and asymmetrical short-circuit faults emerges as an irrefutable mandate^1–3^. Symmetrical faults (SFs), characterized by a balanced distribution of fault currents across the three phases, and asymmetrical faults (AFs), entailing uneven fault impedances among the phases, are formidable challenges that demand astute handling. These fault scenarios can swiftly cascade into disruptions, jeopardizing the uninterrupted operation of DFIG systems and potentially compromising the stability and reliability of the broader power grid network^4,5^. As the integration of renewable energy systems, including wind turbines employing DFIG technology, escalates, the significance of assuring their steadfast operation under fault conditions becomes increasingly pronounced. At the heart of this critical juncture lies the pursuit of seamless solutions that not only mitigate the immediate impacts of faults but also contribute to the longevity and sustainability of renewable energy integration. Effectively protecting DFIG systems and the power grid against these multifaceted fault scenarios transcends mere technical exigencies; it assumes the role of a linchpin for ensuring optimal power generation, quality, and transmission. Additionally, the pervasive global momentum toward sustainable energy sources underscores the urgency of bolstering the resilience of renewable energy systems against potential disruptions^6^.

In view of these dynamics, this paper casts a probing spotlight on the indispensable nature of deploying vigilant and tailored protection strategies for DFIG systems in the face of symmetrical and asymmetrical short-circuit faults. By delving into the nuances of fault detection, characterization, and control, this study contributes to not only the enhanced understanding of these intricate phenomena but also the formulation of advanced strategies to counteract their adverse impacts. Ultimately, the meticulous pursuit of protective mechanisms amalgamated with renewable energy systems will orchestrate a harmonious synergy between evolving technology and the relentless quest for a robust and sustainable energy future^7^. While a fault occurs in the grid connected to a WT equipped with DFIG, two issues of fault detection and limiting the fault effect are momentous^8^. This issue will be done to protect the stator winding and electric converters on the rotor side. Furthermore, under the mentioned advantages, the DFIGs have two elementary problems of system sensitivity to grid disturbances, especially voltage dip and generator output power fluctuations. Since the three-phase stator winding of the generator will be under a direction connected to the grid, a sudden voltage dip in the grid can lead to the current flowing beyond more than the endurance of the RSC and cause serious damage to the mentioned converters^9,10^. In other words, the voltage dips of the output terminals induce large counter-electromotive forces in the rotor, and this leads to a strong transient current passing through the rotor and an increase in the DC-link voltage in the power converter, resulting in damage to the electronic power converter^11^. Numerous researches have been presented (in the form of review papers), to provide methods to increase the DFIG low voltage ride-through capability (LVRTC). These methods will be categorized into two classifications, hardware (passive) and software (active). Hardware methods by adding extra equipment include reactive power injection equipment such as capacitor banks or energy storage equipment; it can provide current limiting conditions in the RSC for fault conditions. On the other hand, in software techniques, the issue of overcoming the LVRT problem is solved by designing a suitable control system for existing converters or wind turbine blade-pitch control systems^12,13^.

Focuses on modeling and transient stability analysis of type-3 WTs using mathematical techniques like singular perturbation and Lyapunov methods in^14^. The goal is to enhance the understanding of transient stability in WT systems. The study proposes a mathematical model that incorporates the dynamic behavior of wind turbines, taking into account singular perturbation techniques to simplify the model's equations. This simplification aids in analyzing transient stability under various operating conditions. Lyapunov methods are then employed to assess the stability of the system over time. The article's contribution lies in its utilization of advanced mathematical tools to provide insights into the transient behavior of Type-3 WTs. Discusses the seismic performance analysis of a WT with a monopile foundation subjected to sea ice effects in^15^. A simple numerical method is employed to evaluate the WT's behavior under such conditions. The study investigates the impact of sea ice-induced vibrations on the structural integrity of the monopile foundation and the overall performance of the wind turbine. The results obtained from the numerical simulations offer insights into the potential risks associated with sea ice-induced vibrations and suggest potential mitigation strategies to enhance the seismic performance of wind turbines in icy environments. The following focuses on the construction of simplified models for dynamic analysis of monopile-supported offshore WTs^16^. The objective is to create simplified yet accurate models that can capture the dynamic behavior of wind turbines while reducing computational complexity. The study proposes methods to model the dynamic response of the WT system, taking into account factors such as wave loads, wind loads, and foundation characteristics. By simplifying the models, the authors aim to provide a practical approach for assessing the dynamic behavior and performance of offshore WTs with monopile foundations. Further, addresses the challenge of diagnosing bearing faults in wind turbine gearboxes under actual operating conditions using limited data that may contain noise^17^. The study focuses on developing a fault diagnosis method that can accurately identify bearing faults despite the presence of noise in the data. The authors employ machine learning techniques to train a model that can differentiate between normal gearbox operation and faulty conditions. By labeling data with noise, the study aims to improve the model's robustness in real-world scenarios. The article's significance lies in its application of machine learning for fault diagnosis in WT systems, contributing to the field of condition monitoring and maintenance. In^18^, explores the practical stability analysis of wind power systems through the application of the limit cycle amplitude tracing (LCAT) method. The main objective is to assess the system's stability region in a multi-parameter space, taking into consideration various operating conditions and parameter variations. The LCAT technique involves tracking the amplitude of limit cycles to identify stability boundaries, which allows for a comprehensive understanding of the system's behavior. By analyzing the stability region, the study aims to provide valuable insights into the dynamic behavior of wind power systems and enable better system design and control strategies.

Proposes a wind speed correction method that utilizes a modified hidden Markov model (HMM) to enhance the accuracy of wind power forecasts in^19^. The study addresses the challenge of forecasting wind power production accurately by accounting for the uncertainty and variability of wind speed. The modified HMM takes into consideration historical wind speed data to improve the forecast accuracy by correcting the wind speed predictions. This method has the potential to contribute to more reliable wind power predictions, aiding energy grid operators and stakeholders in optimizing power generation and distribution plans. Focuses on the model predictive current control of nine-phase open-end winding permanent magnet synchronous motors (PMSMs) in^20^. The study introduces an online virtual vector synthesis strategy to enhance the efficiency and performance of the control system. Model predictive control (MPC) techniques are employed to regulate the current output of the motor while considering the unique characteristics of nine-phase PMSMs. The article's contribution lies in the development of a control strategy that combines MPC and virtual vector synthesis, offering improved current regulation and overall motor performance. In^21^, presents a generic carrier-based pulse width modulation (PWM) solution designed for series-end winding PMSM traction systems. The study introduces an adaptive overmodulation scheme to enhance the modulation range and control flexibility of the PWM method. The proposed solution aims to optimize the utilization of the PMSM in traction applications while addressing challenges related to overmodulation and switching losses. By developing an adaptive overmodulation scheme, the article contributes to the advancement of efficient and reliable control strategies for PMSM-based traction systems. Further, delves into the development and analysis of a high-performance solar-driven thermoelectric generator (TEG) system combined with radiative cooling in^22^. The study explores the concept of utilizing radiative cooling alongside solar energy concentration to enhance the efficiency of thermoelectric power generation. The system's performance is likely assessed in terms of energy conversion efficiency, power output, and overall feasibility for passive power generation. This innovative approach addresses the challenges of sustainable energy generation and offers insights into the integration of multiple renewable energy technologies to maximize efficiency and reduce environmental impact.

In^23^, focuses on voltage sag state estimation through a multi-stage approach utilizing an event-deduction model. The study aims to accurately estimate voltage sag states in power systems by analyzing various parameters such as EF (frequency event), EG (sag duration event), and EP (phase angle event). The multi-stage methodology is designed to provide a more comprehensive and accurate representation of voltage sag events, which is crucial for maintaining power quality and system reliability. The article’s contribution lies in its approach to improving the accuracy of voltage sag state estimation, which is essential for effective power system operation and management. Introduces a fast-dynamic phasor estimation algorithm specifically designed for phasor measurement units (PMUs) in power systems in^24^. The study addresses the challenge of accurately estimating phasors in dynamic scenarios while considering DC offset effects. The proposed algorithm aims to provide real-time and accurate phasor measurements, which are essential for power system monitoring, control, and stability assessment. The article’s significance lies in its contribution to improving the accuracy and speed of phasor estimation algorithms, enhancing the reliability and efficiency of PMU applications in power systems. Focuses on the design of a double-side flux modulation permanent magnet (PM) machine intended for servo applications in^25^. The study likely explores the unique characteristics and advantages of the double-side flux modulation technique in PM machines. This technique involves manipulating the flux distribution in the machine's rotor to achieve improved performance characteristics such as torque density and efficiency. The article’s contribution lies in its application of innovative design principles to create a PM machine suitable for servo applications, addressing the demands of precise and dynamic motion control systems. Examines power coupling in a voltage source converter (VSC) system connected to a weak AC grid in^26^. The study likely investigates the challenges associated with coupling power between the converter and the grid under weak grid conditions. The article may propose an improved decoupling control strategy to enhance the converter’s performance and grid stability. By addressing power coupling issues and providing effective control solutions, the article contributes to the understanding and advancement of VSC systems’ integration into weak AC grids, which is essential for reliable power transmission and distribution.

Reference^27^ introduces an innovative approach to flux weakening in five-phase PMSM motors, addressing issues with conventional methods. It considers voltage limits affected by harmonic current control and employs a feed-forward flux weakening algorithm to optimize the current trajectory. Gradient descent ensures stability. Non-linear harmonic current control prevents inverter current limits. Deadbeat current control and space vector PWM generate duty cycles. Successfully applied in a five-phase PMSM, it proves effective. A simultaneous diagnosis method for open-circuit power switch and current sensor faults in grid-connected three-level neutral point clamped inverters is presented in^28^. It uses an adaptive interval sliding mode observer to accurately track three-phase currents while minimizing steady-state resonance. A sensitive faulty phase detection scheme with adaptive thresholding is employed. Fault type identification distinguishes open-circuit faults and sensor faults. Hardware-in-the-loop tests confirm the method's effectiveness and robustness in detecting 12 open-circuit faults and 9 sensor faults.

Reference^29^ introduces a novel sliding mode disturbance observer-based technique for diagnosing demagnetization faults in interior PM (IPM) motors while eliminating stator parameter mismatch impacts. It establishes an IPM motor model accounting for disturbances from PM demagnetization and stator parameter mismatch. A sliding mode disturbance observer identifies disturbances, focusing on flux linkage mismatch. The extracted disturbance is used to determine demagnetization faults and degrees. Experimental validation on two IPM motors confirms the effectiveness of the proposed method. Analyzing SPM motors with shaped magnets and quasi-regular polygon rotor core (QPRC) rotors is challenging due to their unique structure^30^. This article presents a new subdomain method to accurately predict their electromagnetic performance. It segments shaped magnets symmetrically and employs variable rotor core radius for QPRC analysis. A periodic boundary condition accelerates performance prediction, including air-gap flux density, cogging torque, back-EMF, inductance, electromagnetic torque, and unbalanced magnetic force. Applied to a 12-pole/3-phase surface-mounted PM (SPM) motor, the technique's accuracy is confirmed through finite-element analysis and experiments, with calculation speed significantly faster than conventional methods.

This paper addresses the intricate challenge of ensuring WT robustness during fault conditions, a concern for power system experts. To tackle this, SFRT and AFRT control techniques are crucial. The study introduces an innovative SFRT–AFRT strategy named RCO, optimized for the RSC in a DFIG used in WTs. The approach involves attenuating fault components and optimizing the control characteristic ‘*β*’ using a PSO algorithm. This optimizes RSC performance during faults. The proposed method is compared with FO-PID controllers. Through this comparison, the novel approach's effectiveness is comprehensively evaluated, providing insights into its advantages and limitations for fault mitigation. The research focuses on embedding optimized coefficients of the ‘*β*’ element using PSO into the designed objective function, guiding RNSV command parameters via defined constraints. The proposed method lets PSO determine the ‘*β*’ value. The resulting ‘*β*’ optimized coefficients are applied as reference values to compensate for rotor components. The control system comprises a voltage capacity limiter and RNSV optimization for independent AFRT on a 1.9 MW DFIG. MATLAB/SIMULINK simulates the system, examining dynamic behavior during LLL and LL faults (12 Ω and 0 Ω resistance). PSO proves effective in optimizing ‘*β*’, outperforming other algorithms in speed, accuracy, and computational efficiency. Finally, the main contributions of this paper are listed as follows:

- The paper introduces a novel control strategy named RCO, designed specifically for the RSC within a DFIG used in WT systems.
- The proposed RCO strategy optimizes the performance of the RSC under fault conditions through an innovative two-fold approach. It attenuates both positive and negative fault components and optimizes the control characteristic ‘*β*’ using a PSO algorithm.
- The utilization of a PSO algorithm for optimizing the control characteristic ‘*β*’ is a noteworthy innovation. This optimization approach is well-suited for solving complex optimization problems, ensuring effective performance under fault scenarios.
- The paper conducts a comprehensive comparative analysis by juxtaposing the performance of the proposed SFRT–AFRT RCO control technique with FO-PID controllers.
- The simulation results demonstrate the effectiveness of the proposed method in reducing fluctuations during fault conditions, leading to improved stability of the DFIG system.

The rest of the paper is organized as follows. “Modeling of DFIG under ‘SF’ conditions” Section provides the modeling of DFIG under ‘SF’ conditions. “Modeling of DFIG under ‘AF’ conditions” Section provides the modeling of DFIG under ‘AF’ conditions. “The proposed technique” Section presents the description of the proposed technique. “Simulation results and discussion” Section discusses the simulation results, and “Conclusion” Section concludes this paper.

## Modeling of DFIG under ‘SF’ conditions

Modeling a DFIG under ‘SF’ conditions involves analyzing the generator’s behavior during a balanced fault in the power system. A 'SF', also known as a balanced fault, is a fault condition in which all three phases of a power system experience the same fault at the same time^31,32^. This typically occurs due to short circuits or other disturbances in the system^33,34^. When modeling a DFIG under ‘SF’ conditions, the goal is to understand how the generator behaves during a balanced fault where all three phases of the power system experience the same fault simultaneously. This situation usually arises due to short circuits or other disturbances.

The modeling process involves the DFIG electrical aspect as follows:

- *Stator Side* The stator winding of the DFIG is a critical component. During a ‘SF’, equations representing the stator voltage, current, and impedance are employed. These equations consider the stator’s resistance, inductance, and mutual inductance between windings. The fault's impact is factored in by altering the parameters in the equations to simulate the faulted conditions accurately.
- *Rotor Side* The rotor winding is unique due to its connection through slip rings and brushes. Equations are developed to capture the behavior of the rotor current under ‘SF’ conditions. Similar to the stator, rotor resistance, inductance, and mutual inductance are taken into account. These equations reflect the rotor's response to the fault and its effect on the overall system dynamics.

## Modeling of DFIG under ‘AF’ conditions

**Figure 1:**
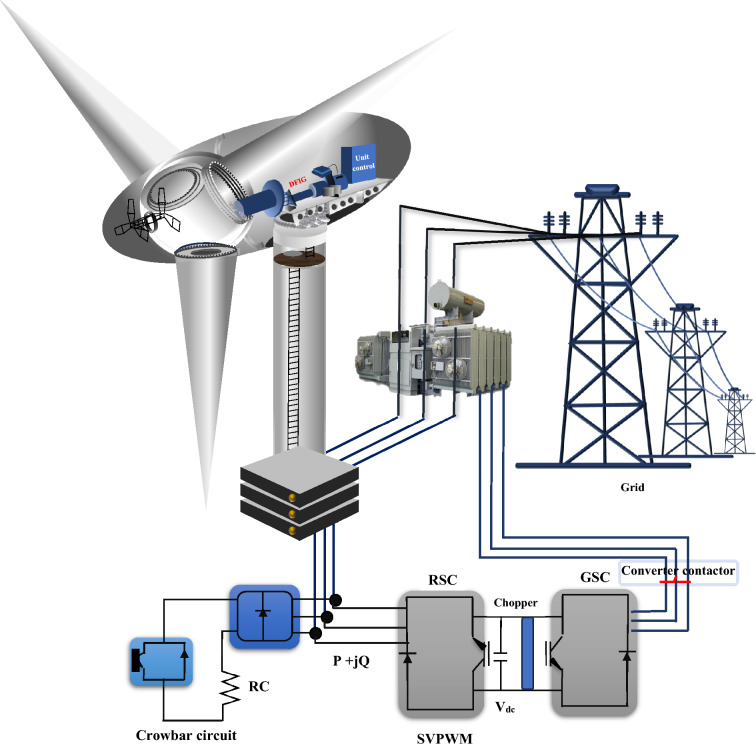
The structure of the DFIG.

A DFIG is an induction machine that (according to Fig. 1), the stator is directly connected to the grid, and the rotor winding has connected to the grid through a pair of back-to-back converters with a common DC link^35–37^. The desired DFIG specifications are presented in Table 1. In this topology, the RSC is a converter, that is, used to supply an excitation voltage to the induction generator rotor windings. The GSC (grid side converter) is a rectifier that keeps the DC bus voltage stable^38,39^. Underneath the standard case, the DFIG mathematical model will be represented based on the voltage and flux linkage vector relations in the PRSRF as follows:

1 $$\left\{ {\begin{array}{*{20}c} {U_{sd - q}^{ + } = R_{s} I_{sd - q}^{ + } + p\Psi_{sd - q}^{ + } + j\omega_{1} \Psi_{sd - q}^{ + } } \\ {U_{rd - q}^{ + } = R_{r} I_{rd - q}^{ + } + p\Psi_{rd - q}^{ + } + j\omega_{slip} \Psi_{rd - q}^{ + } } \\ {\Psi_{sd - q}^{ + } = L_{s} I_{sd - q}^{ + } + L_{m} I_{rd - q}^{ + } } \\ {\Psi_{rd - q}^{ + } = L_{m} I_{sd - q}^{ + } + L_{r} I_{rd - q}^{ + } } \\ \end{array} } \right.$$

where $U_{sd - q}^{ + }$ is the stator voltage vector, $U_{rd - q}^{ + }$ is the rotor voltage vector, $I_{sd - q}^{ + }$ refers to the stator current vector, $I_{rd - q}^{ + }$ is the rotor current vector, $\Psi_{sd - q}^{ + }$ is the stator flux linkage vector, and $\Psi_{rd - q}^{ + }$ is the rotor flux linkage vector, $\omega_{slip} = \omega_{1} - \omega_{r}$ refers to the slip angular frequency amid the PRSRF and the rotor. In the ‘AF’ situations, all the parameters in (1) retain P–S and N–S features defined as:

2 $$F_{d - q}^{ + } = F_{{d - q^{ + } }}^{ + } + F_{{d - q^{ - } }}^{ - } e^{{ - 2\omega_{1} t}}$$

where $F_{{d - q^{ + } }}^{ + }$ refers to the P–S segment in the PRSRF,$F_{{d - q^{ - } }}^{ - }$ refers to the N–S segment in the NRSRF, and $2\omega_{1} t$ is the angle discrepancy between the PRSRF and NRSRF. By replacing (2) with (1), the mathematical sample of the DFIG in the ‘AF’ states is defined as:

3 $$\left\{ {\begin{array}{*{20}c} {U_{{sd - q^{ + } }}^{ + } = R_{s} I_{{sd - q^{ + } }}^{ + } + p\Psi_{{sd - q^{ + } }}^{ + } + j\omega_{1} \Psi_{{sd - q^{ + } }}^{ + } } \\ {U_{{rd - q^{ + } }}^{ + } = R_{r} I_{{rd - q^{ + } }}^{ + } + p\Psi_{{rd - q^{ + } }}^{ + } + j\omega_{slip} \Psi_{{rd - q^{ + } }}^{ + } } \\ {\Psi_{{sd - q^{ + } }}^{ + } = L_{s} I_{{sd - q^{ + } }}^{ + } + L_{m} I_{{sd - q^{ + } }}^{ + } } \\ {\Psi_{{rd - q^{ + } }}^{ + } = L_{m} I_{{rd - q^{ + } }}^{ + } + L_{r} I_{{rd - q^{ + } }}^{ + } } \\ \end{array} } \right.$$

4 $$\left\{ {\begin{array}{*{20}c} {U_{{sd - q^{ - } }}^{ - } = R_{s} I_{{sd - q^{ - } }}^{ - } + p\Psi_{{sd - q^{ - } }}^{ - } - j\omega_{1} \Psi_{{sd - q^{ - } }}^{ - } } \\ {U_{{rd - q^{ - } }}^{ - } = R_{r} I_{{rd - q^{ - } }}^{ - } + p\Psi_{{rd - q^{ - } }}^{ - } + j\omega_{{slip^{ - } }} \Psi_{{rd - q^{ - } }}^{ - } } \\ {\Psi_{{sd - q^{ - } }}^{ - } = L_{s} I_{{sd - q^{ - } }}^{ - } + L_{m} I_{{sd - q^{ - } }}^{ - } } \\ {\Psi_{{rd - q^{ - } }}^{ - } = L_{m} I_{{rd - q^{ - } }}^{ - } + L_{r} I_{{rd - q^{ - } }}^{ - } } \\ \end{array} } \right.$$

where $\omega_{{slip^{ - } }} = - \omega_{1} - \omega_{r}$; further, the relation (3) describes the flux linkage and voltage vector relations of the P-S segment in the PRSRF; also, the relation (4) illustrates the flux linkage and voltage vector relations of the negative ordering segments in the NRSRF. For comfort in the subsequent investigation, (3) and (4) are rewritten as *d*–*q* elements, as illustrated in (5) and (7).

5 $$\left\{ {\begin{array}{*{20}l} {u_{{sd^{ + } }}^{ + } = R_{s} i_{{sd^{ + } }}^{ + } + p\psi_{{sd^{ + } }}^{ + } - \omega_{1} \psi_{{sq^{ + } }}^{ + } } \hfill \\ {u_{{sq^{ + } }}^{ + } = R_{s} i_{{sq^{ + } }}^{ + } + p\psi_{{sq^{ + } }}^{ + } + \omega_{1} \psi_{{sd^{ + } }}^{ + } } \hfill \\ {u_{{rd^{ + } }}^{ + } = R_{r} i_{{rd^{ + } }}^{ + } + p\psi_{{rd^{ + } }}^{ + } - \omega_{slip} \psi_{{rq^{ + } }}^{ + } } \hfill \\ {u_{{rq^{ + } }}^{ + } = R_{r} i_{{rq^{ + } }}^{ + } + p\psi_{{rq^{ + } }}^{ + } + \omega_{slip} \psi_{{rd^{ + } }}^{ + } } \hfill \\ {\psi_{{sd^{ + } }}^{ + } = L_{s} i_{{sd^{ + } }}^{ + } + L_{m} i_{{rd^{ + } }}^{ + } } \hfill \\ {\psi_{{sq^{ + } }}^{ + } = L_{s} i_{{sq^{ + } }}^{ + } + L_{m} i_{{rq^{ + } }}^{ + } } \hfill \\ {\psi_{{rd^{ + } }}^{ + } = L_{m} i_{{sd^{ + } }}^{ + } + L_{r} i_{{rd^{ + } }}^{ + } } \hfill \\ {\psi_{{rq^{ + } }}^{ + } = L_{m} i_{{sq^{ + } }}^{ + } + L_{r} i_{{rq^{ + } }}^{ + } } \hfill \\ \end{array} } \right.$$

in which,

6 $$\left\{ {\begin{array}{*{20}c} { U_{{sd - q^{ + } }}^{ + } = u_{{sd^{ + } }}^{ + } + ju_{{sq^{ + } }}^{ + } } \\ {U_{{rd - q^{ + } }}^{ + } = u_{{rd^{ + } }}^{ + } + ju_{{rq^{ + } }}^{ + } } \\ {I_{{sd - q^{ + } }}^{ + } = i_{{sd^{ + } }}^{ + } + ji_{{sq^{ + } }}^{ + } } \\ {I_{{rd - q^{ + } }}^{ + } = i_{{rd^{ + } }}^{ + } + ji_{{rq^{ + } }}^{ + } } \\ {\Psi_{{sd - q^{ + } }}^{ + } = \psi_{{sd^{ + } }}^{ + } + j\psi_{{sq^{ + } }}^{ + } } \\ {\Psi_{{rd - q^{ + } }}^{ + } = \psi_{{rd^{ + } }}^{ + } + j\psi_{{rq^{ + } }}^{ + } } \\ \end{array} } \right.$$

7 $$\left\{ {\begin{array}{*{20}l} {u_{{sd^{ - } }}^{ - } = R_{s} i_{{sd^{ - } }}^{ - } + p\psi_{{sd^{ - } }}^{ - } + \omega_{1} \psi_{{sq^{ - } }}^{ - } } \hfill \\ {u_{{sq^{ - } }}^{ - } = R_{s} i_{{sq^{ + } }}^{ - } + p\psi_{{sq^{ - } }}^{ - } - \omega_{1} \psi_{{sd^{ - } }}^{ - } } \hfill \\ {u_{{rd^{ - } }}^{ - } = R_{r} i_{{rd^{ - } }}^{ - } + p\psi_{{rd^{ - } }}^{ - } - \omega_{{slip^{ - } }} \psi_{{rq^{ - } }}^{ - } } \hfill \\ {u_{{rq^{ - } }}^{ - } = R_{r} i_{{rq^{ - } }}^{ - } + p\psi_{{rq^{ - } }}^{ - } + \omega_{{slip^{ - } }} \psi_{{rd^{ - } }}^{ - } } \hfill \\ {\psi_{{sd^{ - } }}^{ - } = L_{s} i_{{sd^{ - } }}^{ - } + L_{m} i_{{rd^{ - } }}^{ - } } \hfill \\ {\psi_{{sq^{ - } }}^{ - } = L_{s} i_{{sq^{ - } }}^{ - } + L_{m} i_{{rq^{ - } }}^{ - } } \hfill \\ {\psi_{{rd^{ - } }}^{ - } = L_{m} i_{{sd^{ - } }}^{ - } + L_{r} i_{{rd^{ - } }}^{ - } } \hfill \\ {\psi_{{rq^{ - } }}^{ - } = L_{m} i_{{sq^{ - } }}^{ - } + L_{r} i_{{rq^{ - } }}^{ - } } \hfill \\ \end{array} } \right.$$

in which,

8 $$\left\{ {\begin{array}{*{20}c} { U_{{sd - q^{ - } }}^{ - } = u_{{sd^{ - } }}^{ - } + ju_{{sq^{ - } }}^{ - } } \\ {U_{{rd - q^{ - } }}^{ - } = u_{{rd^{ - } }}^{ - } + ju_{{rq^{ - } }}^{ - } } \\ {I_{{sd - q^{ - } }}^{ - } = i_{{sd^{ - } }}^{ - } + ji_{{sq^{ - } }}^{ - } } \\ {I_{{rd - q^{ - } }}^{ - } = i_{{rd^{ - } }}^{ - } + ji_{{rq^{ - } }}^{ - } } \\ {\Psi_{{sd - q^{ - } }}^{ - } = \psi_{{sd^{ - } }}^{ - } + j\psi_{{sq^{ - } }}^{ - } } \\ {\Psi_{{rd - q^{ - } }}^{ - } = \psi_{{rd^{ - } }}^{ - } + j\psi_{{rq^{ - } }}^{ - } } \\ \end{array} } \right.$$

**Table 1:** The parameters of the DFIG.

Parameter	Symbol	Best score
Rated capacity	$S_{N}$	1.9 MVA
Stator/rotor windings turn ratio	–	0.44
Mutual inductance	$L_{m}$	3.95 pu
Stator self-induction	$L_{s}$	4.65 pu
Rotor self-induction	$L_{r}$	4.25 pu
Stator resistance	$R_{s}$	0.0072 pu
Rotor resistance	$R_{r}$	0.0063 pu
DC-link capacitor	$C_{dc}$	12,000 μF

## The proposed technique

### FO-PID technique

A Fractional-Order proportional–integral–derivative (FO-PID) controller is an advanced control methodology that extends the conventional PID controller by integrating principles from fractional calculus. Fractional calculus deals with derivatives and integrals of non-integer order, allowing the FO-PID controller to address intricate system dynamics that standard integer-order controllers might struggle to capture effectively^40–44^. The FO-PID controller is structured as follows:

9 $$C\left( s \right) = K_{p} + \frac{{K_{i} }}{{S^{\alpha } }} + K_{d} S^{\beta }$$

where $C\left( s \right)$ represents the transfer function of the FO-PID controller.$K_{p}$,$K_{i}$ and $K_{d}$ correspond to the proportional, integral, and derivative gains, respectively. Furthermore, *S* the complex frequency variable. In the following, *α* and *β* signify the fractional orders of the integral and derivative terms, respectively.

The introduction of fractional-order terms provides the FO-PID controller with the ability to address systems featuring non-integer dynamics. The fractional-order integral term $\left( {\frac{1}{{S^{\alpha } }}} \right)$ is particularly significant as it accommodates processes with memory effects and gradual dynamics. Conversely, the fractional-order derivative term ($S^{\beta }$) is effective in handling systems with anticipatory behaviors and rapid transitions. Selecting appropriate values for *α* and *β* is contingent upon the specific characteristics of the controlled system. It's worth noting that the design and tuning of FO-PID controllers can be more intricate than their integer-order counterparts due to the inclusion of fractional-order parameters^45–47^. Determining suitable values for $K_{p}$,$K_{i}$, $K_{d}$, *α*, and *β* often necessitates the application of optimization techniques and system identification methodologies. FO-PID controllers exhibit notable potential across a range of applications, particularly in scenarios characterized by long time delays, anomalous diffusion, and non-integer order dynamics. Their capability to model intricate system behaviors has generated substantial interest in leveraging FO-PID controllers to enhance control accuracy and system stability within diverse engineering domains. This innovative approach showcases how fractional calculus principles can be harnessed to advance control strategies and cater to the complexities of modern engineering challenges.

### RCO technique

**Figure 2:**
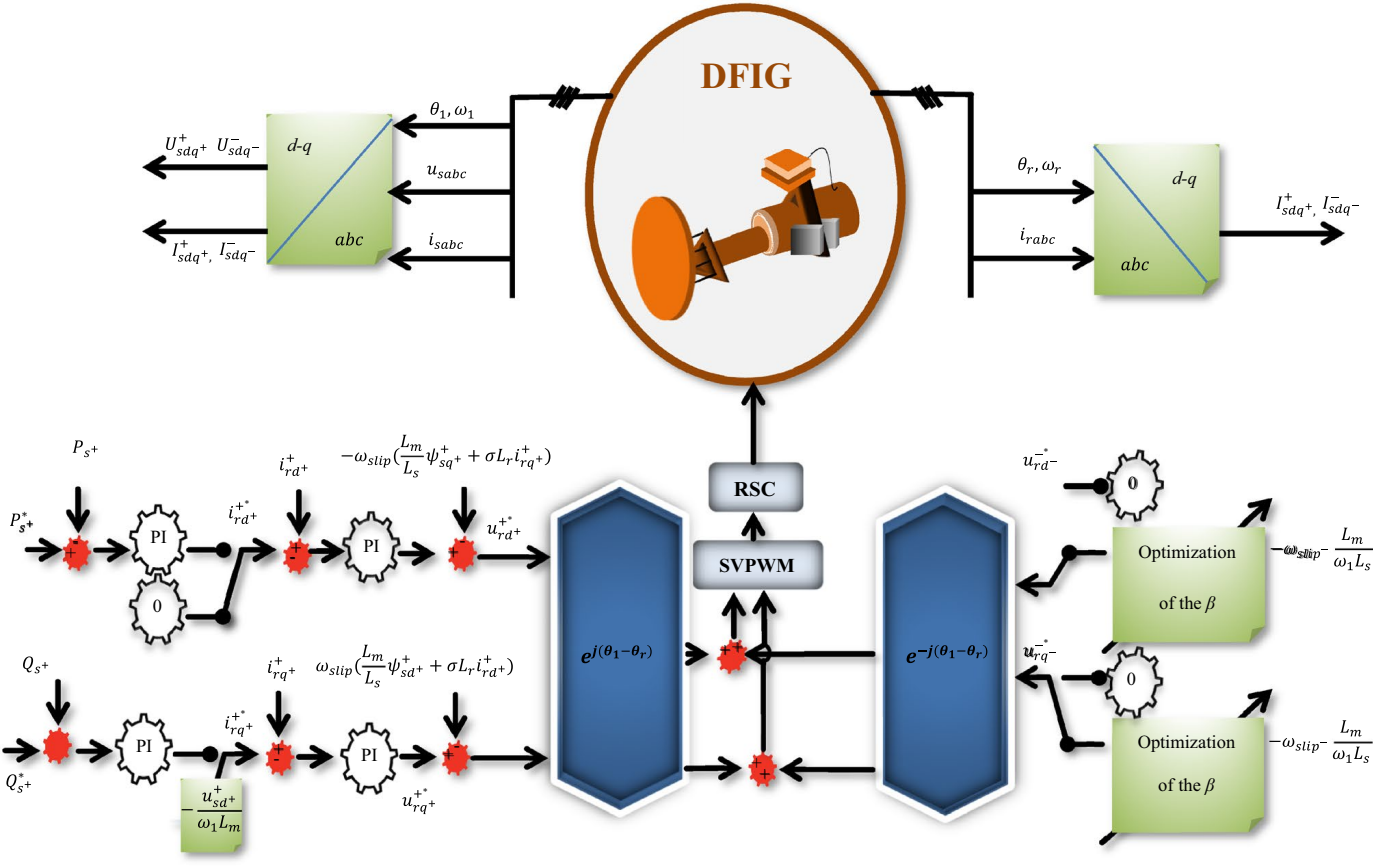
The proposed controller for RSC.

**Figure 3:**
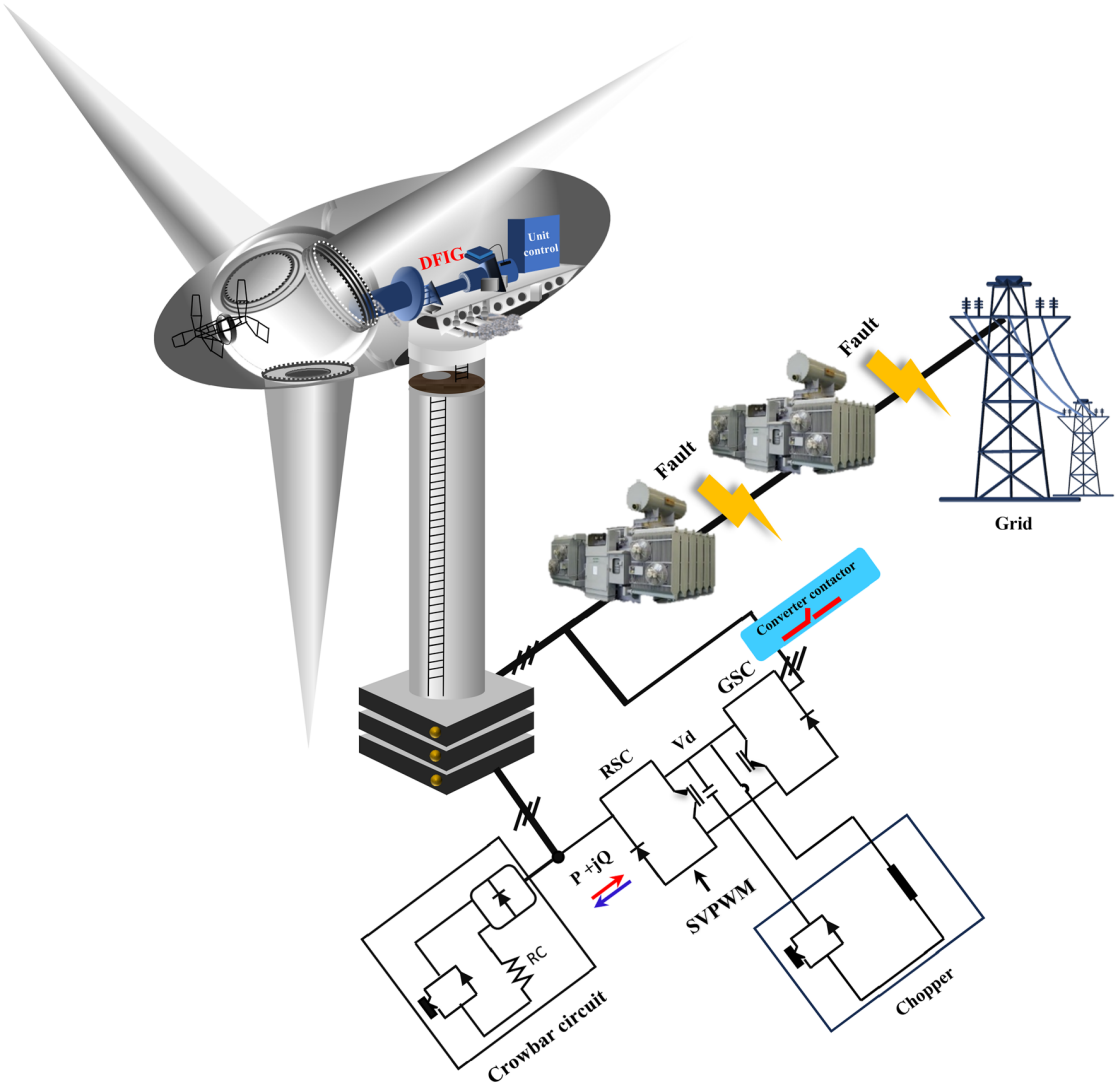
The fault condition modeling.

In this section, the objective functions will include RNSV components that have been programmed. The optimized coefficients of the *β* element will be embedded in the designed objective function by the PSO method and will be set as the required RNSV command parameters by taking the constraints determined for the problem. Therefore, in the proposed method, the PSO will determine the commensurate characteristic *β* based on the defined objective function. Finally, the reference values with the ‘*β*’ optimized coefficients to compensate for the rotor required components are applied. Also, producing switching pulses to the RSC is provided by the pulse width modulation interface. The proposed controller for RSC to manage the FRT condition is shown in Fig. 2. Further, the fault conditions modeling is presented in Fig. 3.

### PSO method

**Figure 4:**
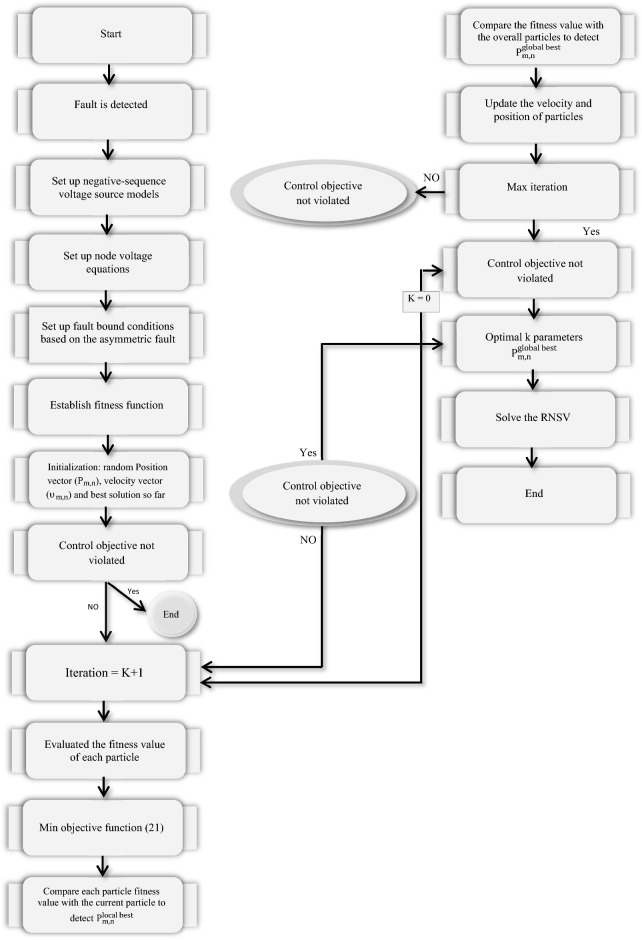
The flowchart of the proposed technique based on the PSO algorithm.

The PSO is a community-based optimization algorithm motivated by the social behavior of bird immigration and displacement^8,48–52^. In this algorithm, each particle describes a possible answer, and it flies via a multidimensional examination area to see the optimal solution by using information from its personal best place and the best position found by the swarm. A flowchart of the steps for implementing the desired technique based on the PSO method is presented in Fig. 4.

10 $$\upsilon_{m,n}^{new} = w \times \upsilon_{m,n}^{old} + {\Gamma }_{1} \times r_{1} \times \left( {P_{m,n}^{local best} - P_{m,n}^{old} } \right) + {\Gamma }_{2} \times r_{2} \times \left( {P_{m,n}^{global best} - P_{m,n}^{old} } \right)$$

11 $$P_{m,n}^{new} = P_{m,n}^{old} + \upsilon_{m,n}^{new}$$

where $\upsilon_{m,n}$ is particle velocity, $P_{m,n}$ is particle variables, ${\Gamma }_{2} {\Gamma }_{1}$ are learning factors, $r_{1} { }r_{2}$ is independent random numbers with uniform distribution, $P_{m,n}^{local best}$ is the best local answer and $P_{m,n}^{global best}$ will be the global best answer. Also, the selected parameters of the PSO are recorded in Table 2.

**Table 2:** Specification of the PSO algorithm.

Parameter	Variable	Set-value
Particle number	*P*	55
Iterations	*N*	250
Cognition learning rate	$\Gamma_{1}$	1.2
Social learning rate	$\Gamma_{2}$	1.2
Min and Max inertia weight	*w*	0.4–0.7
Random numbers	$r_{1} { }r_{2}$	[0,1]

## Simulation results and discussion

### Case I: LLL fault (SF)

**Figure 5:**
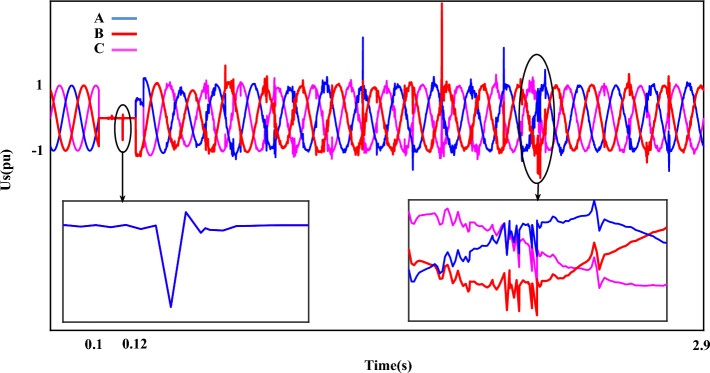
The stator-voltage of DFIG under LLL fault condition.

**Figure 6:**
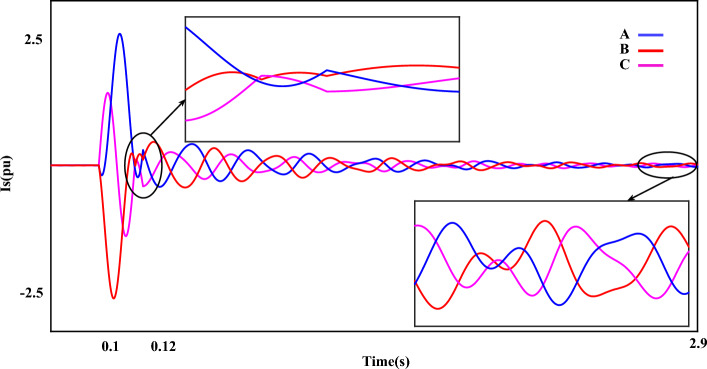
The stator-current of DFIG under LLL fault condition.

Figure 5 shows the generator $U_{s}$ during a SF. Fluctuations and instabilities of voltage in two parts can be easily detected in a magnified form. The fault gaped when the fault occurs is well known in the first part. After the fault, although the measured value of the voltage parameter is within the nominal value, disturbances are still seen in the voltage component. Figure 6 shows the generator $I_{s}$ during a ‘SF’. The effect of a short circuit fault is clearly visible in the stator current. The measured value of current in fault cycles is more than 2.5 pu. Next, as it is clear, although the value of the rotor current is in a certain size, the error fluctuations are still clearly visible in the stator current component. The measured THD value for the stator current component in this plot is more than 14%.

**Figure 7:**
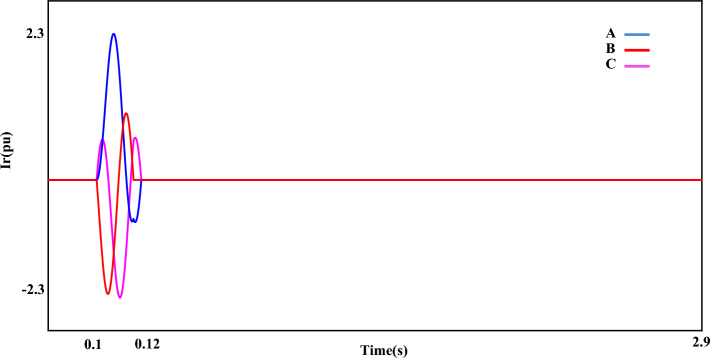
The rotor-current of DFIG under LLL fault condition.

**Figure 8:**
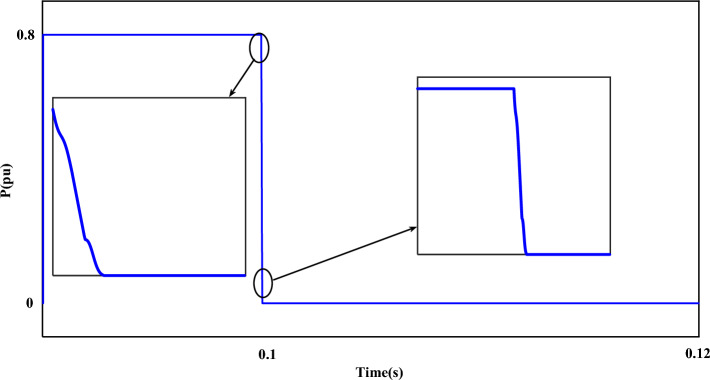
The P component of the grid under LLL fault condition.

**Figure 9:**
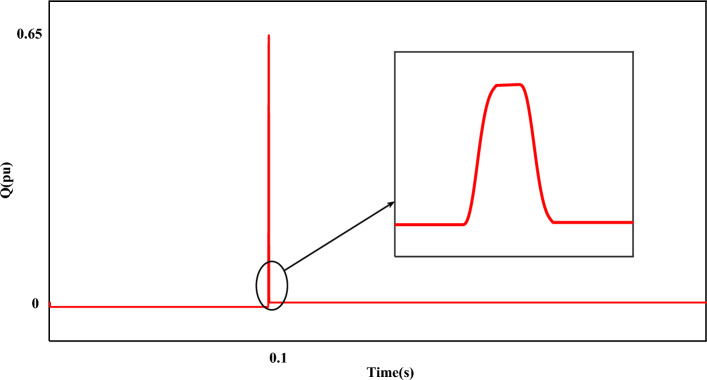
The Q component of the grid under LLL fault condition.

**Figure 10:**
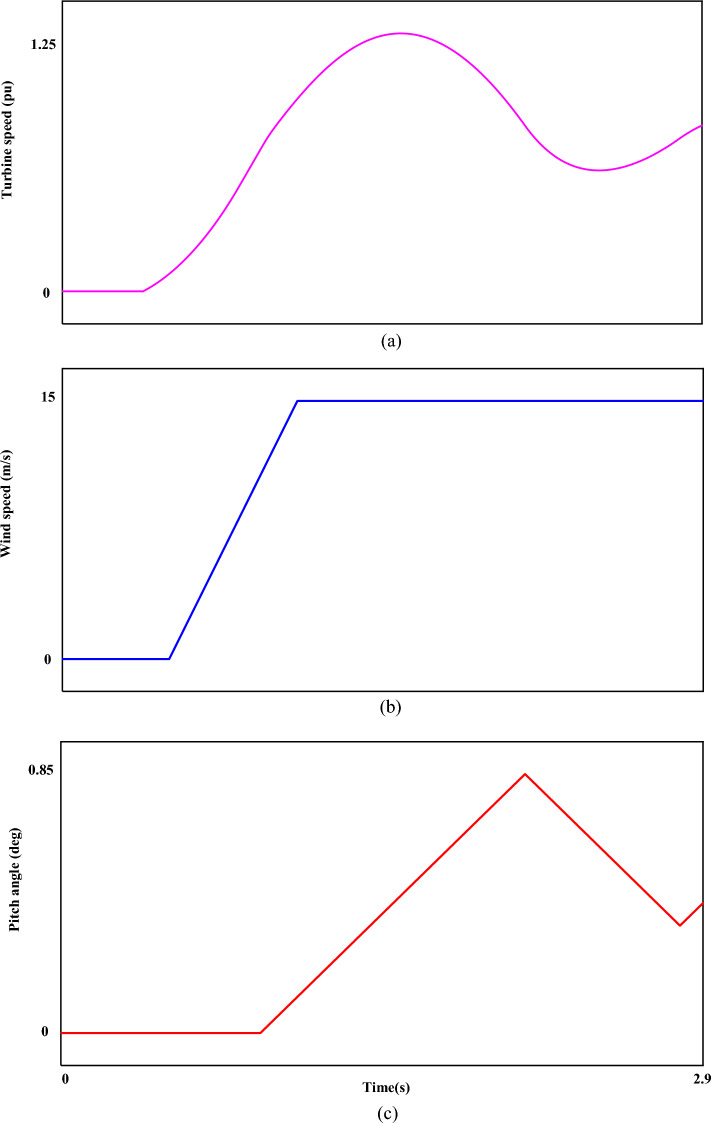
The turbine parameters. (**a**) turbine speed, (**b**) wind speed, (**c**) pitch angle.

Figure 7 shows the fault current ($I_{r}$). The value of the fault current component ($I_{r}$) is 2.5 pu. which is shown separately in the mentioned plot (Fig. 7). The disturbance of each phase can be clearly displayed in the fault condition. Figure 8 shows the measured value of the power component (P). Based on the desired plot, the power of the system is practically unavailable when a fault occurs and decreases from 0.8 pu to 0. By using the zoom feature, the change value of the P component is clearly visible in Fig. 8. The reactive power of the network, like the P component, has a relatively strong reaction based on the plot shown in Fig. 9. The value of Q when the fault occurs is recorded as 0.65 pu. Of course, this issue is predictable for fault modes. Figure 10 shows turbine parameters including turbine speed, wind speed and pitch angle, respectively.

### Case II: LL fault (AF)

**Figure 11:**
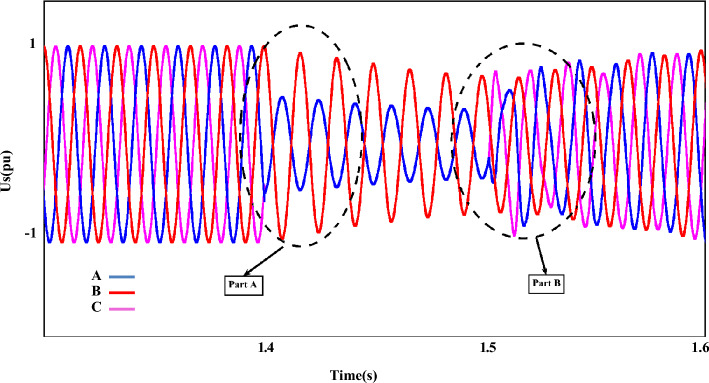
The stator-voltage of DFIG under LL fault condition.

**Figure 12:**
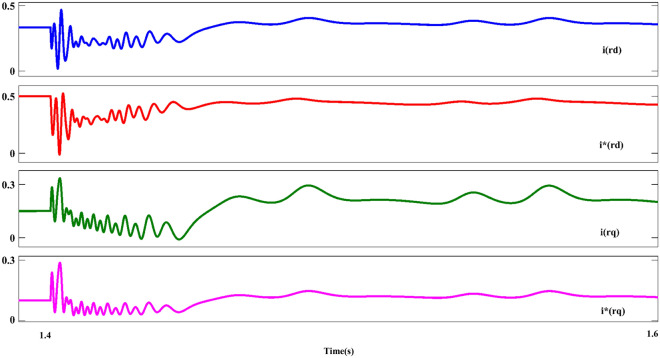
The rotor d-q axis currents for positive-sequence under LL fault condition.

**Figure 13:**
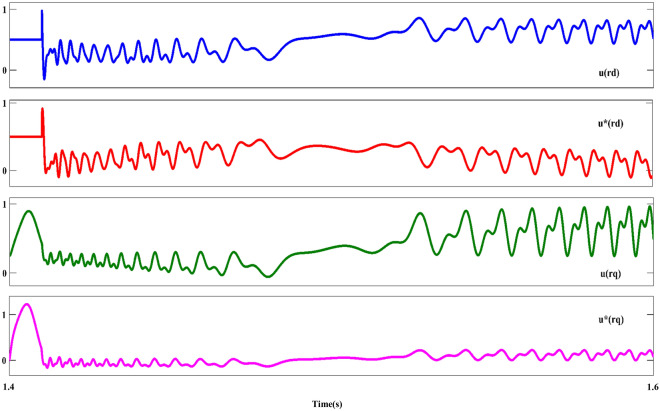
The rotor d-q axis voltages for negative-sequence under LL fault condition.

Figure 11 shows the $U_{{s_{abc} }}$ component; As can be seen in this plot, the asymmetrical of the dips as is the evident component ($U_{{s_{abc} }}$) is after the occurrence of the fault. In the following, the behavior of the stator-voltage during the occurrence of a fault and the onset of AFRT conditions is presented in part ‘A’. Also, considering the faulted phases (a and c) have the same amplitude, for this reason, the mentioned issue is remarked in part ‘B’ of Fig. 11. The reference values of the RPSC ($i_{{rd^{ + } }}^{{ +^{*} }}$ and $i_{{rq^{ + } }}^{{ +^{*} }}$) are computed, as shown in Fig. 12. By examining the mentioned components, the improvement of current operation conditions can be clearly recognized. In the following, the amplitude of the fluctuations of the $i_{{rd^{ + } }}^{ + }$ component in $i_{{rd^{ + } }}^{{ +^{*} }}$ has been reduced by using the first part of the RCO technique. The greatest improvement in operating conditions is related to the $i_{{rq^{ + } }}^{ + }$ component, which is well damped in the positive component using the proposed technique. Further, according to the value set for the reference parameter, the oscillation range will be reduced to zero value. However, the operating conditions after FRT (within a limited period) it's accompanied by an increased range but cannot be ignored of the proposed method (first part of RCO) performance. Figure 13 show voltage reference parameters of the system, including $u_{{rq^{ - } }}^{ - } ,u_{{rq^{ - } }}^{ - } , u_{{rq^{ - } }}^{{ -^{*} }} {\text{and}} u_{{rd^{ - } }}^{{ -^{*} }}$ components. These parameters also have an overshoot like the previous sequence when the fault occurs, and the FRT conditions start in the first cycle, and then by utilizing the RNSC limitation, the overshoot of the components is overcome, and finally, each of the factors converges.

### Case II: LL fault (AF)

Figure 11 shows the $U_{{s_{abc} }}$ component; As can be seen in this plot, the asymmetrical of the dips as is the evident component ($U_{{s_{abc} }}$) is after the occurrence of the fault. In the following, the behavior of the stator-voltage during the occurrence of a fault and the onset of AFRT conditions is presented in part ‘A’. Also, considering the faulted phases (a and c) have the same amplitude, for this reason, the mentioned issue is remarked in part ‘B’ of Fig. 11. The reference values of the RPSC ($i_{{rd^{ + } }}^{{ +^{*} }}$ and $i_{{rq^{ + } }}^{{ +^{*} }}$) are computed, as shown in Fig. 12. By examining the mentioned components, the improvement of current operation conditions can be clearly recognized. In the following, the amplitude of the fluctuations of the $i_{{rd^{ + } }}^{ + }$ component in $i_{{rd^{ + } }}^{{ +^{*} }}$ has been reduced by using the first part of the RCO technique.

The greatest improvement in operating conditions is related to the $i_{{rq^{ + } }}^{ + }$ component, which is well damped in the positive component using the proposed technique. Further, according to the value set for the reference parameter, the oscillation range will be reduced to zero value. However, the operating conditions after FRT (within a limited period) it's accompanied by an increased range but cannot be ignored of the proposed method (first part of RCO) performance.

Figure 13 show voltage reference parameters of the system, including $u_{{rq^{ - } }}^{ - } ,u_{{rq^{ - } }}^{ - } , u_{{rq^{ - } }}^{{ -^{*} }} {\text{and}} u_{{rd^{ - } }}^{{ -^{*} }}$ components. These parameters also have an overshoot like the previous sequence when the fault occurs, and the FRT conditions start in the first cycle, and then by utilizing the RNSC limitation, the overshoot of the components is overcome, and finally, each of the factors converges.

**Figure 14:**
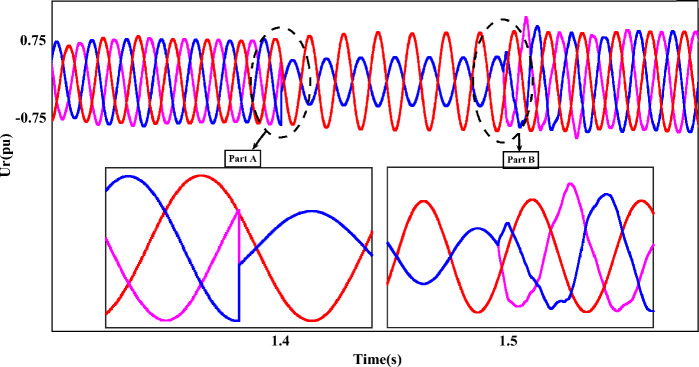
The rotor-voltages of DFIG under LL fault condition.

**Figure 15:**
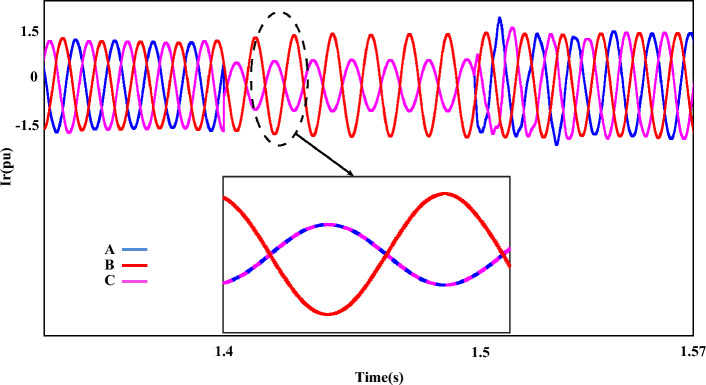
The rotor-currents of DFIG under LL fault condition.

It is worth mentioning that the output of each component is accompanied by fluctuations and has a range of 0.5 pu, which will be reduced to 0.23 pu by implementing the proposed method. Figure 14 shows the output of $U_{r}$, which provides the operating conditions of this component during FRT. Amplitude changes of two phases A and C are clearly magnified in two parts. In the first part, the drop of the rotor voltage component up to 0.3 pu has completely changed the operating conditions. On the other hand, after FRT, although the operating conditions of $U_{r}$ have improved to a suitable extent, voltage fluctuations and disturbances are still visible, the issue mentioned in the enlarged second part is quite clear. In this plot, the output amplitude of the $U_{r}$ component is 0.75 pu. Figure 15 represents the rotor-current ($i_{r}$), and the dynamic behavior of this component is the same as in the previous plot ($U_{r}$).

**Figure 16:**
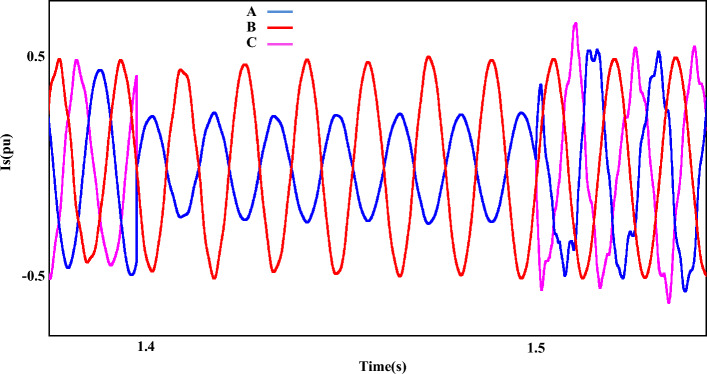
The stator-currents of DFIG under LL fault condition.

Figure 16 shows the stator current output ($i_{s}$) during AF. Here, the fault created on two phases A and C occurs and the measured current value for $i_{s}$ component is 0.25 pu when the fault occurs. By passing through the fault, the content of the waveform is not favorable and some parameters such as disturbances, imbalance, etc. are clearly observed in it. Furthermore, the total harmonic distortion value for the stator current component ($i_{s}$) is more than 12%.

**Figure 17:**
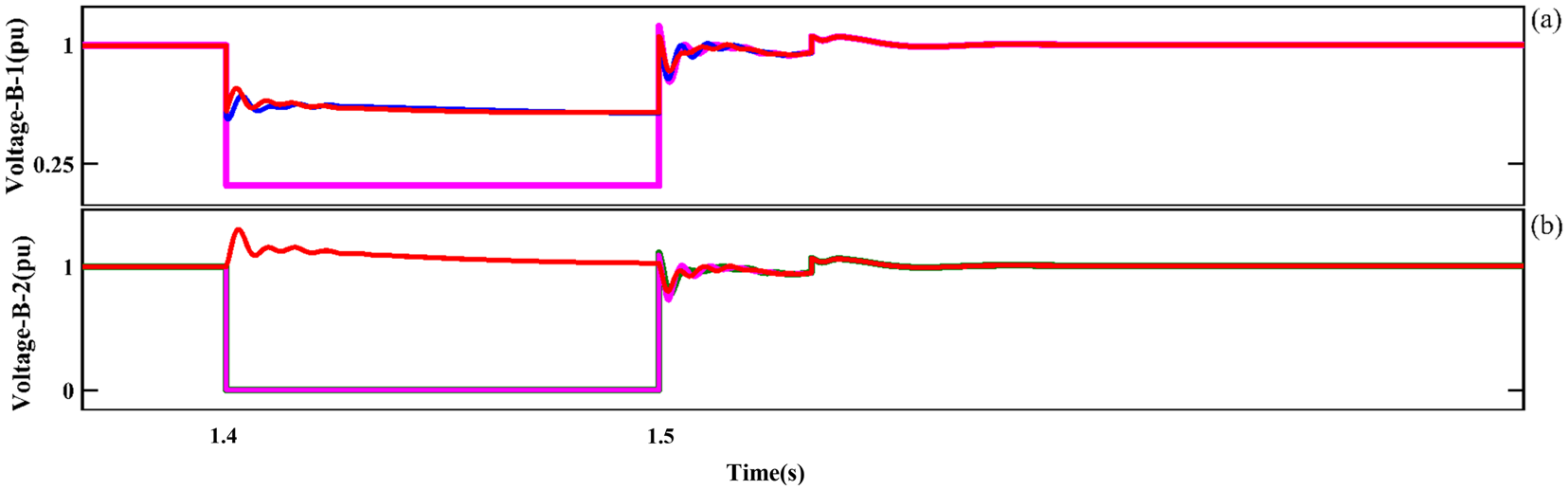
The three adjacent busbars of the grid side under LL fault conditions. (**a**) First busbar output, (**b**) Second busbar output.

Further, the voltages, active power, and reactive power components behaviors of the two adjacent busbars (Bus1 and Bus2) located on the grid side will be investigated under LL fault conditions. Figure 17 shows the voltages of the two adjacent busbars on the grid side separately (under LL fault conditions). Next, the measured value of the network voltage component (phases A, B and C) when AF occurs for the first Bus is 0.65 pu, 0.65 pu and 0.15 pu respectively. Also, for the second Bus, which is adjacent to the location of the fault, the measured values of 0.01 pu, 1 pu and 0.01 pu were recorded for the mentioned phases, respectively.

**Figure 18:**
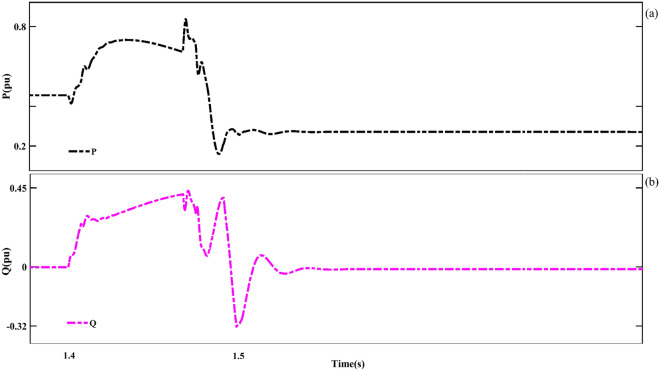
The P and Q components of the grid under LL fault conditions.

**Figure 19:**
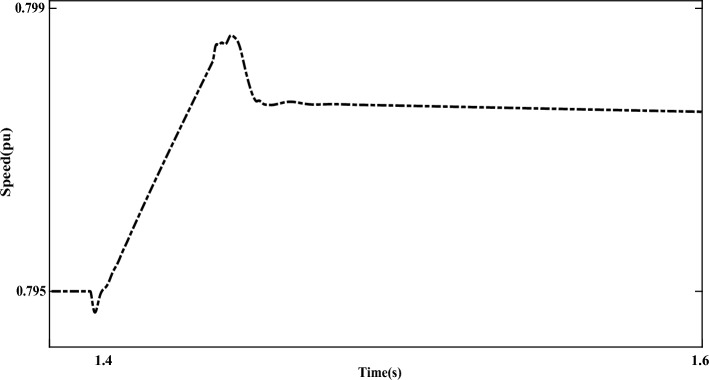
The turbine speed under LL fault conditions.

Figure 18 shows the P and Q components of the grid under LL fault conditions. The measured values of each of these components in fault conditions are 0.85 pu and − 0.32 pu, respectively. Further, the turbine speed under LL fault conditions is shown in Fig. 19. The measured turbine speed value increases to 0.798pu when the fault occurs. After the fault, the turbine speed will decrease.

### Case III: RCO (second part) and FO-PID techniques in LL fault

**Figure 20:**
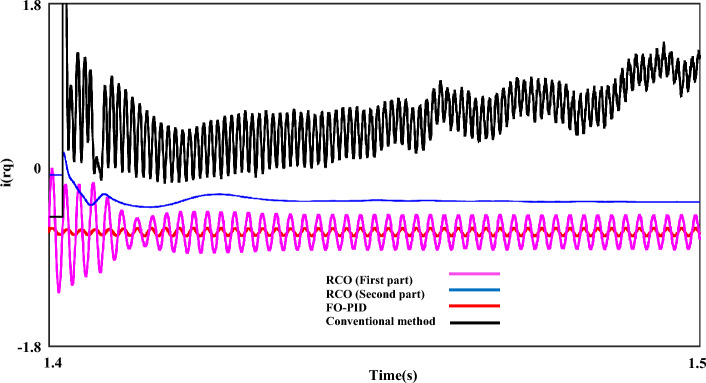
The rotor q-axis currents for negative-sequence under LL fault, with and without proposed techniques (RCO (first and second order), FO-PID).

In the final section, the output dynamic behavior of each of the RNSC and RNSV parameters in the q-axis in the LL fault with 0 Ω resistance is presented. Figure 20 is the output waveform of the $i_{rq}$ component in four working modes including conventional, the RCO (first order), the RCO (second order), and FO-PID, respectively.

In the mentioned figure, the black output shows the operation of the $i_{rq}$ component with the conventional control mode. The fluctuation range of the $i_{rq}$ parameter in this mode has also reached 1pu, and in the continuation of the work, in addition to the fact that the fluctuations of the system are not damped, it can be seen that the operating conditions have become much more difficult, and finally the measured value of $i_{rq}$ fluctuates from 1.2 pu to 1.4 pu. Next, the worst operating conditions are related to the RCO-first order mode, which, although it has fluctuations with a range of 0.6pu-0.8pu, the system's fluctuations range is constant and compared to the conditions with the conventional mode, the system has better operation. The red output shows the operating conditions of the system in the FO-PID mode, which has a relatively good performance. Finally, the blue output shows the operation of the system in the RSO-second order mode. It can be seen that by using this mode, except for the first few cycles, which have fluctuations, the output of $i_{rq}$ is completely smooth.

**Figure 21:**
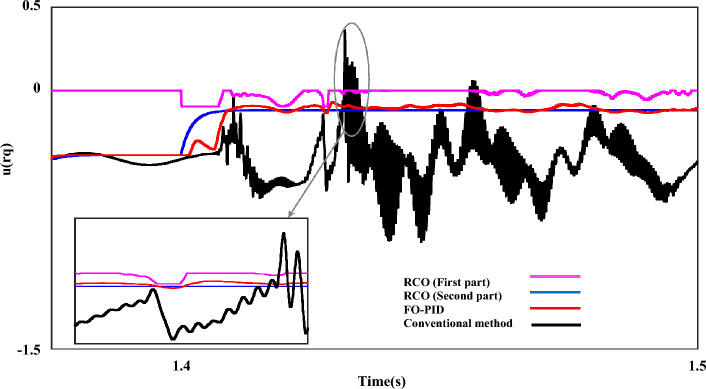
The rotor q-axis voltages for negative-sequence under LL fault, with and without proposed techniques (RCO (first and second order), FO-PID).

**Figure 22:**
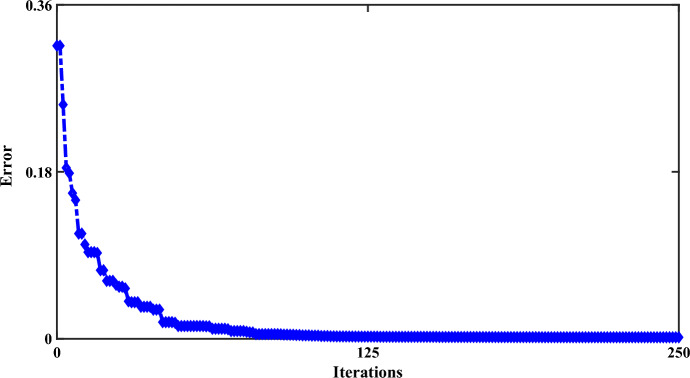
The proposed algorithm (PSO) iteration diagram.

Figure 21 shows the $u_{rq}$ component with four different working modes. Like the example of the $i_{rq}$ component, in the conventional mode, the system experiences the worst operating conditions, and the best performance of the system is related to the RCO-first order, FO-PID and the RCO-second order. Figure 22 shows the iteration output of the employed algorithm. The PSO algorithm is effective in solving many optimization problems, including those related to the optimization of the characteristic ‘*β*’. The performance of these algorithms has been evaluated based on several metrics, including convergence speed, accuracy, and computational efficiency. The PSO algorithm has been found to outperform the other algorithms in several ways. This means that PSO can find the optimal value of the characteristic* β* in fewer iterations than the other algorithms.

Finally, in terms of computational efficiency, PSO can find the optimal value of the coefficient *β* with fewer computational resources than FO-PID. Additionally, the computational efficiency of PSO can be demonstrated by comparing the amount of computational resources required by each algorithm to find the optimal value of the *β* coefficient. In the following, the values of the ‘*β*’ factor with initial default values for this coefficient are presented in Table 3.

**Table 3:** Selected coefficients of ‘*β’* characteristic.

Procedure	LLL fault		ll fault	
	$\beta_{d}$	$\beta_{q}$	$\beta_{d}$	$\beta_{q}$
Initial input values	5.852	10.391	11.254	19.625
PSO	0.815	0.941	1.157	1.858

Here, the purpose of presenting this research is to investigate the dynamic behavior of system voltage and current parameters in the FRT conditions in the RSC section of a DFIG. As presented in the previous sections, the fault conditions considered for this study include the LLL scenario and the LL faults. The proposed method to reduce the fluctuations caused by the mentioned faults includes two steps. The first step includes the reduction of RNSC and RNSV based on the definition of the reference values (first order of the RCO) and finally, the second stage is based on the optimization of the ‘*β*’ characteristic to determine and set up the best output value of the controller(second order of the RCO). The obtained results show the improvement of the output conditions of the desired components by inserting the proposed method. In this study, the proposed method to address the critical challenge of ensuring the robust operation of WTs during fault conditions. By introducing the innovative SFRT–AFRT control methodology called RCO and optimizing the control characteristic ‘*β*’ using a PSO algorithm, they aim to enhance the performance of the RSC within a DFIG in wind turbine systems. This suggested approach includes several advantages:

- The SFRT–AFRT RCO control strategy is designed to attenuate both positive and negative fault components, ensuring that the WT system remains stable and reliable even during adverse grid conditions. This is crucial for maintaining grid reliability and preventing cascading failures.
- The PSO algorithm utilization for optimizing the control characteristic '*β*' is a significant advantage. PSO is known for its effectiveness in solving complex optimization problems, ensuring that the RSC performs optimally under fault scenarios.
- In this study, a thorough comparative analysis by comparing the performance of the proposed SFRT–AFRT RCO control technique with FO-PID controllers has been presented. This evaluation provides valuable insights into the superiority of the novel approach, helping to identify its advantages and limitations.
- The simulation results demonstrate that the proposed control method effectively reduces fluctuations during fault conditions, leading to improved DFIG stability in WTs. This enhanced stability is vital for preventing grid disturbances and ensuring uninterrupted electricity supply.
- The paper also highlights the economic viability of the proposed control method, suggesting its suitability for addressing broader power network issues, such as power quality. This implies that the approach can have a positive impact on the overall efficiency and reliability of the power grid.

## Conclusion

This study proposed a control system consisting of a voltage capacity limiter and optimization of the RNSV item on the RSC section to control and support independent SFRT and AFRT on a 1.9 MW DFIG system. The challenge in this study is to analyze the dynamic behavior of the proposed control method in the face of LLL and LL faults with 12 Ω and 0 Ω fault resistance occurring on the transmission line. The simulation results showed that the proposed control method effectively controlled and supported the DFIG system during SFRT and AFRT in both LLL and LL fault conditions. The proposed method to reduce the fluctuations caused by the mentioned faults includes two steps. The first step includes the reduction of RNSC and RNSV based on the definition of the reference values (first order of the RCO) and finally, the second stage is based on the optimization of the ‘*β*’ characteristic to determine and set up the best output value of the controller(second order of the RCO). The simulation results showed that the proposed method significantly reduced the fluctuations in the system parameters during FRT conditions and improved the stability of the DFIG system.

## Data Availability

The datasets used and/or analyzed during the current study available from the corresponding author on reasonable request.
